# Effects of Metalinguistic Awareness on Reading Comprehension and the Mediator Role of Reading Fluency from Grades 2 to 4

**DOI:** 10.1371/journal.pone.0114417

**Published:** 2015-03-23

**Authors:** Liping Li, Xinchun Wu

**Affiliations:** 1 School of Psychology, Beijing Normal University, Beijing, China; 2 School of Teachers Education, Shanxi Normal University, Linfen, China; University of Leicester, UNITED KINGDOM

## Abstract

**Purpose:**

This study examined the contribution of metalinguistic awareness including morphological awareness, phonological awareness and orthographical awareness to reading comprehension, and the role of reading fluency as a mediator of the effects of metalinguistic awareness on reading comprehension from grades 2 to 4.

**Methods:**

Four hundred and fifteen elementary students in China mainland were administered a test battery that included measures of morphological awareness, phonological awareness, orthographical awareness, reading fluency, reading comprehension and IQ. Hierarchical regression and structural equation models (SEM) were used to analyze the data.

**Results:**

Morphological awareness uniquely explained 9%, 10% and 13% variance of reading comprehension respectively from grade 2 to grade 4, however, phonological awareness and orthographical awareness did not contribute to reading comprehension; Reading fluency partially mediated the effect of morphological awareness on reading comprehension in grades 2-4.

**Conclusions:**

These findings indicated that reading fluency and morphological awareness should be facilitated in the Chinese instruction. Morphological awareness played an important role in Chinese reading and affected reading comprehension in grades 2 to 4; Reading fluency was a significant link between morphological awareness and reading comprehension in grades 2-4.

## Introduction

Reading comprehension is a complex processing [1], and different components of metalinguistic awareness are useful for reading comprehension [2]. A number of studies have provided evidence that reading comprehension can be predicted by metalinguistic awareness [3,4,5,6] and reading fluency [7]. Metalinguistic awareness is the ability to analyze, think about, or manipulate language as an object separate from its meaning in or out of context [8]. Phonological awareness, morphological awareness and orthographical awareness have usually been observed as metalinguistic awareness which is regarded to have special importance for reading [9]. Reading fluency refers to read with speed, accuracy, and prosody [10,11]. Furthermore, previous researches confirmed significant associations between metalinguistic awareness and reading fluency [10,12]. For example, phonological knowledge was the precursor of reading fluency [13,14,15], and phonological awareness was a valid and reliable predictor of reading fluency [16].

From the theoretical views, reading fluency may influence reading comprehension. The Lexical Quality Hypothesis [17] and Automatic Theory [18] in reading suggested that if the processing of sub-skills component required much attention, then most cognitive resources were expended on word decoding and low-level processing, leaving few cognitive resources available for other higher-level component skills, such as reading comprehension, drawing inferences and integrating information. However, if the processing of sub-skills component became automatic, higher-level processing of reading could receive more attention and resources. Thus, the accurate and quick reading of words would improve reading comprehension.

From a multidimensional view of reading fluency, metalinguistic awareness may influence reading fluency. Reading fluency was the result of many sub-processes which involved phonological, orthographic, and morphological processes [10,11]. The processing of low-level cognitive skills (phonological awareness, morphological awareness and orthographical awareness) was an essential part to reading fluency and influenced the speed of reading. Previous findings mostly focused on the relationship between reading fluency and reading comprehension, and the relationship of cognitive skills and reading fluency was neglected. Not only the theoretical views but also empirical studies proved the relationship between metalinguistic awareness, reading fluency, and reading comprehension. In the following section, we reviewed previous research.

### Metalinguistic Awareness and Reading Comprehension

Prior researches showed that phonological awareness [3,19,20], morphological awareness [4,1] and orthographical awareness [21] could influence reading. A large body of literature had focused on the relationship between metalinguistic awareness and word reading [12,22,23], because phonology, orthography and morphology were observed as three major lexical constituents [24,25]. Normal reading relied on activating word representation so that words could be identified accurately [26], thus the components of lexicon was indispensable for reading. Few studies had focused on the relationship between metalinguistic awareness and text reading comprehension, especially the relationships among phonological awareness, orthographical awareness and reading comprehension in Chinese because reading comprehension was observed as a high-level and complex process that processed the various lexical constituents.

Given the “morphosyllabic” nature of the Chinese writing system [3,27], the Chinese researches contained relatively more information about the role of morphological awareness on reading comprehension than that of phonological awareness and orthographical awareness. Morphological awareness generally refers to children’s ability to perceive and manipulate the smallest unit of word meaning [28,29,30]. In either alphabetic language or non-alphabetic language [5,31,32], morphological awareness uniquely contributed to reading comprehension. Reading acquisition involved in the meaning-processing [28], and morphology was the minor unit of meaning, so morphology was manipulated in reading. Because there are many compound words which are made up of two or more morphemes [33,34], homophones and homographs [34] in Chinese, the ability to manipulate and distinguish morphemes well is helpful for understanding Chinese words, thereby benefits to text comprehension. Morphological awareness might be important not only for word reading but also for reading comprehension for Chinese students.

There were few attempts to investigate whether phonological awareness and orthographical awareness influenced the high-level and ultimate outcome of reading comprehension. It might due to phonological and orthographic processing involving sound and visual aspects of the word, rather than just the semantics. Chinese is relatively semantically transparent and phonologically unreliable at the word and subword levels [35,36]. Phonological processing skill remained important for reading comprehension, because morphological skills and phonological skills were intrinsic to language, and both were usually coexist [31]. Thus, phonological awareness, the ability to identify and manipulate speech sounds, might affect Chinese reading comprehension. The tasks of orthographical awareness required children to make judgments about whether the target word was in the written word [37] and orthographical awareness explained unique variance in reading for grades 1–3 [38]. Chinese written character has a square shape mapping onto the morphemes [3] and orthographic structure improved morphological awareness [39]. So, phonological and orthographical awareness could play a certain role in Chinese reading comprehension.

However, the roles of cognitive skills are not equally important for different grade levels, and the most powerful cognitive predictors for reading vary for different grades [12]. Prior study showed that the contribution of phonological awareness decreased with the grade level for languages of Hungarian, Dutch and Portuguese [40]. For Chinese, a dynamic developmental pattern of metalinguistic skills underpinning word reading development was revealed [35], but it is unclear how the role of metalinguistic awareness on Chinese reading comprehension changes as a function of grade.

### The mediator of reading fluency

Metalinguistic awareness as the sub-processing skill may influence the high-level processing of reading comprehension directly or via a certain route [41,42], but there are not many studies focusing on the mechanism of metalinguistic awareness on reading comprehension. A few studies [1,41,42] have begun to explore the mechanisms of morphological awareness and reading comprehension. For example, for Spanish-speaking minority learners, passage reading fluency mediated the effect of morphological awareness on reading comprehension in learning English as a second language [1]. However, orthographical awareness was not included in that study. In this study, the mechanisms of phonological awareness and orthographical awareness influencing reading comprehension will be investigated in addition to morphological awareness.

Some researchers suggested [10,11,43] that fluency involved in reading accurately at a minimal rate, with prosody which was with appropriate expression or intonation coupled with the maintenance of meaning [43]. The majority of studies about reading fluency had focused on word-level reading fluency [12], and found oral reading fluency was usually a predictor of reading comprehension in alphabetic language studies [14,44,45,46]. However, reading fluency included not only oral reading fluency but silent reading fluency [44]. Silent and connected-text reading fluency was paid little attention [31], which was the primary mode of reading for proficient readers. So, this current study tried to explore the role of silent and connected-text reading fluency in the context of metalinguistic awareness and reading comprehension. In this study, fluency was conceptualized as speed, accuracy, and deep understanding. Prosody was excluded because prosody mainly included the rise and falls of pitch, rhythm and stress in oral language [43], however, silent text reading fluency in the current study was administered.

At present, the relationship of metalinguistic awareness and reading fluency has not gotten enough attention. Morphological knowledge would impact reading fluency. A morphologically complex word could facilitate recognition of a stem word, and eye movement study showed that fixation times were shorter for targets that followed a morphologically related prime than a control word [47]. Prior study [48] also investigated native Hebrew speakers in fifth grade and found a significant relationship between their performance on inflectional morphological tasks and passage reading fluency, controlling for phonological awareness and memory. Semantic exercises that focused on the meaning of word facilitated the development of reading fluency [49]. If reading was rapid without constructing meaning, it was merely fake reading [50]. In Chinese, the task of morphological production significantly explained variance of reading fluency in grades 4 and 6 [12]. Chinese is a logographic language [3,27] and is more transparent on the semantic aspect than the phonological one [35]. In addition, morphological awareness was the core of Chinese reading comprehension [31] and may be the core of high-quality lexical representation [4]. The ability to be conscious of and manipulate morphology and morphological structure affected the quality of verbal representation. In Chinese reading, many words are formed by the learned characters and morphology, so awareness of morphology is more important than in English. Thus, accessing the character meaning quickly and automatically could be a prerequisite for reading comprehension.

In Chinese, the prior study [12] showed that phoneme awareness contributed to silent reading fluency significantly in grade 2, because students in low grades relied mostly on phoneme decoding when reading during the earlier phase of reading development. However, the eye movement research [51] showed that good phonemic awareness in second grade did not contribute to good silent reading fluency. A research on dyslexic children [52] showed that there was not a significant relationship between phonological awareness and oral text reading fluency. No consensus had been reached on the relationship between phonological awareness and reading fluency. In addition, reading fluency in the above findings was studied as a unique independent variable. Then, what is the role of phonological awareness in the context of reading fluency and reading comprehension? This question has not yet been answered.

The processing of script characters also included orthography in addition to morphology and phonology. According to the Orthographic Depth Hypothesis [53], shallow orthography was easy to decode and read, but decoding was very slow in deep orthography. Orthographic skills and reading fluency were positively interrelated among skilled and less-skilled students [54], and orthographic analogies training yielded improvements in the reading speed of target words [55]. Without well-specified orthographic representations, students would rely on elaborate and time-consuming decoding instead of a more efficient direct word-reading strategy. Orthographic factors explained performance of some processes that underlie reading fluency in Arabic [56]. Orthography created word-specific knowledge, linking visual and verbal material [55], and orthographic awareness affected to decode words automatically into phonemes. However, Chinese orthography is not as shallow as Arabic. Does orthographical awareness contribute to the speed of Chinese reading? This question will be explored in this study.

According to the Lexical Quality Hypothesis and Automatic Theory, the automatic and quick processing of sub-skills can save more resources to allocate to reading comprehension. However, the empirical results of the studies on the relationship between reading fluency and reading comprehension were not consistent with each other. Many empirical studies demonstrated that reading fluency influenced reading comprehension [7,44,45,57,58]. However, other studies showed that reading fluency could not contribute to it [59]. In Chinese, what is the relationship between reading fluency and reading comprehension? The present study attempted to explore this relationship in the context of cognitive skills.

Though the above researches and theoretical reasons suggested that three metalinguistic skills (phonological awareness, morphological awareness and orthographical awareness) might contribute to reading comprehension and influenced reading fluency [1,13,41,42,], very few empirical studies had investigated this relationship in the context of integrated cognitive factors. So, there was a need to investigate the extent to which the relationship between metalinguistic awareness and reading comprehension was explained by reading fluency. According to the stages of reading [60], the stage of learning to read was in the low and intermediate grades. During this stage, children’s cognitive skills improve quickly, and the role of cognitive skills on reading comprehension may change with grade level. Thus, we chose grades 2 to 4 in the current study, and sought to investigate: 1) whether metalinguistic awareness (phonological awareness, morphological awareness, and orthographical awareness) would uniquely predict reading comprehension from grades 2 to 4; and 2) whether reading fluency mediated the relationship between metalinguistic awareness and reading comprehension from grades 2 to 4.

In this study, structural equation modeling (SEM) was used to determine whether the mediated model yielded a significant contribution to reading comprehension in the elementary schools and whether the mediated model would change with grade level. The advantage of using SEM over traditional regression approaches is that SEM uses latent constructs and is less influenced by measurement errors.

## Method

### Participants

The participants were 415 children (212 boys and 203 girls) in grades 2 to 4 from two primary schools in Shanxi province, China. There were 135 second-graders (62 boys, 73 girls), 142 third-graders (68 boys, 74 girls) and 138 fourth-graders (82 boys, 56 girls). Their mean ages were 90.50 months (*SD* = 5.53), 103.43 months (*SD* = 5.13) and 115.38 months (*SD* = 6.76), respectively. According to teachers’ reports, there were no children in the samples with severely impaired reading or linguistic awareness.

### Measures

#### Morphological awareness

Homophone awareness: In this task, children were firstly presented orally with a morpheme in a compound word. They were then asked to name another word with this same morpheme to confirm that they know the target morpheme and its pronunciation. Next, the children were asked to think of one or more words, using a homophone of this morpheme, within 30 seconds. For example, the target morpheme /yue4/月(moon) in /yue4 liang4/月亮(moon) was presented to the children. The children were asked to produce as many words as possible with the same /yue4/pronunciation, such as /yue4/阅in/yue4du2/阅读(read). Children were encouraged to name as many words as they could. The children produced one new homophone character to record 1 score. Thus, there was no maximum score. The number of correct words with different homophone morphemes that the children produced constituted the score of this task. This task comprised 2 practice items and 12 test items. The internal consistent coefficient α was 0.90.

Homograph awareness: In this task, the experimenter orally presented a two-syllable Chinese word to each child individually. Within that two-morpheme word, one morpheme was identified. The child was then asked to produce two words with the target morpheme. One word contained the morpheme that had the same meaning as the target morpheme. The other word contained the morpheme that had a meaning different from its original meaning. However, both morphemes were identical in pronunciation. For example, when the experimenter gave the target word /shou3biao3/手表(watch), the child was asked to produce a new word with the morpheme /biao3/, in which the /biao3/ had the same meaning as it did in /shou3biao3/. One acceptable answer would be /zhong1biao3/钟表(clock). The meanings of /biao3/ in /shou3biao3/ and /zhong1biao3/ were same, and both were about the time. The child was also asked to say a word that included the morpheme /biao3/ in which its meaning was different from that in /shou3biao3/. An example was /biao3xian4/表现(performance). The meanings of /biao3/ in / shou3biao3/ and /biao3/ in /biao3xian4/ were different because the former was about the time and the latter was about the behavior. All items consisted of real words. There were 2 practice items and 12 test items. A correct response received 1 score. The total score was 24. The internal consistent coefficient α was 0.88.

Compound word production: Following Liu and McBride-Chang (2010) [30], the children were asked to produce a novel word in response to an orally presented question/scenario. The children were encouraged to produce the novel word that could most properly express the meaning conveyed by the question/scenario. Only when they could retrieve the critical morphemes and combine them according to the specific structure indicated by the question/scenario could children be considered to have produced the model answer. The critical and related morphemes coming from the given question/scenario were considered as correct morphemes. The rules of applying morphemes to structure Chinese words were the correct morphological structure. In this test, children applied these correct morphemes to form new words according to the morphological structures. There were 8 practice items and 20 test items. The test items were divided into two parts, and the correct answer of the first part included two morphemes, a total of 12 items. The correct answer of the second part included three morphemes, a total of 8 items. The responses of the children were scored on a scale of 0–3 according to two aspects: structure and morpheme. The score 3 was given for completely correct structure and concise morphemes, the score 2 for correct structure and partly concise morphemes, the score 1 for correct structure but not concise morphemes, and the score 0 for completely wrong structure and morpheme. For example, “用叶子做成的盘子叫什么?”(what do we name the dish made of leaf?). A score of 2 would be given for “ye4pan2叶盘”(leaf-dish), a score of 1 for “ye4zi3pan2叶子盘(adding a redundant suffix “zi3” to leaf), or ye4pan2zi3叶盘子(adding a redundant suffix “zi3” to dish)”, and a score of 0 for “unrelated morpheme”. Two scorers rated children’ answers independently. The inter-rater reliability was 0.95. If there were discrepancies for two scorers, discrepancies were resolved in discussion. Testing stopped when children failed to answer five consecutive items. The internal consistent coefficient α of this test was 0.90。

#### Phonological awareness

Syllable deletion: This task was administered to children orally. They were asked to take away one syllable of monosyllabic phrases. There were a total 4 practice items and 10 test items, including 4 two-syllable and 6 three-syllable phrases. And 4 items deleted the beginning, 4 items deleted the end and 2 items deleted the middle, respectively. For example, “what was left if we didn’t say /yu2/鱼 in /ya2shu1yu2/牙书鱼”. The correct answer was / ya2shu1/牙书. A score of 1 was marked to each correct answer. The maximum possible score was 10. The internal consistent coefficient α was 0.71.

Phoneme deletion: The experimenter orally presented a syllable. The children were asked to produce a new syllable by taking away the target phoneme from a monosyllabic Chinese word. There were 6 practice items and 12 test items. There were three sets of 4 items, deleting the beginning, middle and end, respectively. For example, “what was left when /ch/ was deleted in /cha1/?” The answer was /a1/. Each correct response received a score of 1. The maximum possible score was 12. The internal consistent coefficient α was 0.85.

#### Orthographical awareness

The test involved four ways of structuring characters: 15 items of position error, which contained illegal positions but legal components of Chinese; 15 items of disordered stroke, which contained disordered strokes; 15 items of radical error, which had incorrect components but legal positions; 45 pseudo characters, which consisted of legal components of Chinese in legal positions, but these characters were artificial characters and did not exist. This test contained a total of 90 items. The children were asked to draw “√” when judging the target character to be a character and draw “×” when judging the target character not to be a character. Each correct answer received a score of 1. The maximum possible score was 45 without scoring the pseudo characters because pseudo characters were intended to be fillers. The internal consistent coefficient α was 0.79.

#### IQ

Children’s non-verbal IQ was evaluated by a standardized Chinese version of Raven’s Progressive Matrices [61]. In this task, a pattern with a missing part was presented, and children were asked to choose one appropriate option from the options to complete the target pattern. Formal testing procedures were followed as outlined in the manual. Being the controller factor, the raw value of IQ was analyzed. The internal consistent coefficient α was 0.93.

#### Reading fluency

Silent reading fluency: This task [12,44] served as the indicator of reading fluency. Children were required to judge and mark whether these sentences were correct during a 3-minute interval. The meanings of the sentences were familiar for children in mainland. In the pilot study, the accurate rate to judge the information in each item for children in Grade 1 was higher than 97% if measured without a time limit. For example, “天安门在北京(√)” (Tiananmen square lies in Beijing). “老虎喜欢吃青草(×)” (The tiger likes to eat grass). There were 3 practice items and 100 experimental items. The sentences of 100 trials were ranked from short to long across the test. The number of characters in the marked sentences which children made in 3-minute interval was calculated. Total scores were calculated by counting the number of correct characters responses and subtracting the number of incorrect characters responses (to control for guessing). The internal consistency reliability was 0.97.

#### Reading comprehension

This task examined the ability of understanding, integrating and referring information. From grade 2 to grade 4, children were asked to read a passage and answered 18, 12 and 11 questions respectively, including multiple-choice items and constructed-response items. For example, there was a story of *Prince Nezha's Triumph Against Dragon King* (selected form *Journey to the West*). One question was “According to the passage, which one of Nezha’s characters is not correct? Why? A) He is a boy who is brave. B) He has two weapons. C) He does good things for people. D) He is a bad person.” Total scores of grade 2, grade 3, and grade 4 were 18、16、15, respectively. The internal consistent coefficient α was 0.77、0.62、0.56, respectively. Two scorers made the scores for the constructed-response items simultaneously and independently. The consistent coefficient for two scorers was between 0.64~0.85 in grade 3, and between 0.61~0.74 in grade 4.

### Procedure

This research was implemented in the middle of the autumn semester, at the beginning of November. All measures were administered to the children by trained experimenters and were completed in five different days to avoid fatigue. Orthographical awareness, silent reading fluency and reading comprehension were administered to children in groups, whereas other tests were administered individually in a quiet room in two schools.

### Ethics statement

This study was approved by the Research Ethics Committee of Beijing Normal University. School principals, classroom teachers and parental consents were obtained. The participant teachers and parents supported our study very much. When we got the parental questionnaire which wasn’t analyzed in the present study, parents were informed and signed the consents.

## Results

### Data issues and descriptive statistics

The data were examined for missing data and outliers, which were checked by the regression interpolation method. The resulting skewness and kurtosis values showed that the data were normally distributed. Descriptive statistics were calculated, including means, standard deviations and range of scores in Table 1. All raw data were calculated.

**Table 1:** Descriptive Statistics for Observed Variables by Grades.

measure	G2	G3	G4	*F*	pairwise comparison
	*M* (*SD*)	*M* (*SD*)	*M* (*SD*)		
IQ	38.09(9.40)	40.44(7.51)	43.31(5.87)	15.70	G2<G3<G4
SD	8.84(1.42)	8.98(1.18)	9.77(.42)	28.86	G2 = G3<G4
PD	9.13(3.05)	9.34(2.16)	10.43(1.78)	12.01	G2 = G3<G4
HPA	10.23(5.86)	12.08(4.59)	16.96(5.72)	56.79	G2<G3<G4
CWP	17.16(11.49)	21.97(12.11)	30.72(11.55)	47.10	G2<G3<G4
HGA	10.18(3.64)	11.52(3.54)	15.49(3.50)	82.43	G2<G3<G4
OA	37.96(5.12)	39.61(4.24)	40.74(4.02)	13.34	G2<G3 = G4
SRF	371.44(225.66)	520.54(230.73)	681.89(227.43)	63.32	G2<G3<G4
RC	8.69(3.58)	6.70(2.69)	8.10(2.53)		

Note. IQ = Raven’s Standard Progressive Matrices (raw scores); SD = Syllable deletion; PD = Phoneme deletion; HPA = Homophone awareness; CWP = Compound word production; HGA = Homograph awareness; OA = orthographical awareness; SRF = Silent reading fluency; RC = Reading comprehension. Equal sign indicated non-significant difference, and less-than symbol indicated *p* <. 05 or less.

To investigate the effect of grade level, we conducted two MANOVA analyses and two one-way ANOVAs: one for each of phonological awareness, morphological awareness, reading fluency, and orthographical awareness. In each analysis, grade was the between-subjects factor. For phonological awareness, the three grades differed, Wilks’λ = .87, *F* (4, 822) = 15.46, *p* <. 00. The second MANOVA with morphological awareness as the dependent variable also revealed a significant effect of grade, Wilks’λ = .66, *F* (6, 820) = 32.01, *p* <. 00. One-way ANOVA performed with reading fluency similarly and revealed significant grade difference, *F* (2, 412) = 63.32, *p* <. 00. The second one-way ANOVA was conducted to test the effect of grade for orthographical awareness, *F* (2,412) = 13.34, *p* <. 00. Generally, skills on all measures improved gradually across grade levels. Pairwise comparison was shown in Table 1. However, the second graders and the third graders did not significantly differ in syllable deletion and phoneme deletion. The fourth graders did not significantly outperform the third graders on measures of orthographical awareness.

Tables 2 and 3 presented a summary of the correlations among all measures for three grades. Morphological awareness measures were the strongest correlates of reading fluency and reading comprehension in all grades. Orthographical awareness was the strongest correlate of reading fluency and reading comprehension in grades 2, but not in grades 3–4. Phonological awareness correlated with reading fluency and reading comprehension in grades 2–3, but not in grade 4. Reading fluency and reading comprehension were significantly correlated in three grades, and the correlations of both were 0.58、0.48、0.29 respectively. The correlations between reading fluency and reading comprehension decreased with grade level.

**Table 2:** Correlations for Observed Variables for Grade 2.

measure	1	2	3	4	5	6	7	8	9
1.IQ	-								
2.SD	.20*	-							
3.PD	.45***	.38***	-						
4.HPA	.37***	.21*	.24**	-					
5.CWP	.37***	.34***	.35***	.43***	-				
6.HGA	.24**	.42***	.34***	.40***	.46***	-			
7.OA	.23**	.16	.12	.24**	.25**	.17	-		
8.SRF	.27**	.27**	.25**	.42***	.40***	.52***	.35***	-	
9.RC	.39***	.35***	.31***	.31***	.32***	.46***	.21*	.58***	-

Note. IQ = Raven’s Standard Progressive Matrices (raw scores); SD = Syllable deletion; PD = Phoneme deletion; HPA = Homophone awareness; CWP = Compound word production; HGA = Homograph awareness; OA = orthographical awareness; SRF = Silent reading fluency; RC = Reading comprehension.

*** *p*<. 001;

** p<. 01;

* p <. 05.

**Table 3:** Correlations for Observed Variables for Grades 3 and 4.

measure	1	2	3	4	5	6	7	8	9
1.IQ	-	-.01	.09	.12	.16	.31***	.07	.08	.32***
2.SD	.15	-	.12	-.09	.14	.05	.05	-.08	-.05
3.PD	.21*	.32***	-	.18*	.23**	.23**	.13	-.07	.13
4.HPA	.19*	.18*	.18*	-	.25**	.41***	.03	.07	.18*
5.CWP	.27**	.30***	.18*	.36***	-	.45***	.06	.16	.39***
6.HGA	.31***	.25**	.36***	.37***	.38***	-	.13	.21**	.40***
7.OA	-.01	.07	.11	.04	.00	.05	-	.03	.10
8.SRF	.23**	.26**	.30***	.29***	.33***	.41***	.03	-	.29***
9.RC	.47***	.27**	.30***	.38***	.28**	.46***	.01	.48***	-

Note. Correlations above the diagonal were for grade 4, and correlations below the diagonal were for grade 3. IQ = Raven’s Standard Progressive Matrices (raw scores); SD = Syllable deletion; PD = Phoneme deletion; HPA = Homophone awareness; CWP = Compound word production; HGA = Homograph awareness; OA = orthographical awareness; SRF = Silent reading fluency; RC = Reading comprehension.

*** *p*<. 001;

** p<. 01;

* p <. 05.

### Contributions of metalinguistic awareness to reading comprehension

The predictor variables in this study were related to 7 tasks, which fell into four constructs: phonological awareness, morphological awareness, orthographical awareness and reading fluency. As displayed in Table 4, three hierarchical regression models were calculated to test the unique contributions of orthographical awareness, phonological awareness and morphological awareness to reading comprehension. In the first model, IQ was entered first, followed by morphological awareness measures and phonological awareness measures. The orthographical awareness was entered in the final step. In the second model, displayed in the middle panel of Table 4, the phonological awareness measures were entered in the final step after the morphological awareness measures and the orthographical awareness. In the third model, displayed in the lower panel of Table 4, the morphological awareness measures were entered in the final step after the phonological awareness measures and the orthographical awareness. For grades 2–4, only morphological awareness uniquely predicted the variance of reading comprehension.

**Table 4:** Hierarchical regression explaining reading comprehension from one of three cognitive skills with IQ and other variables controlled.

		G2		G3		G4	
step	variables	*R* ^*2*^	*ΔR* ^*2*^	*R* ^*2*^	*ΔR* ^*2*^	*R* ^*2*^	*ΔR* ^*2*^
1	IQ	.16	.16***	.22	.22***	.14	.14***
2	MA, PA	.34	.18***	.39	.17***	.29	.15***
3	OA	.35	.01	.39	.00	.29	.00
2	MA,OA	.33	.17***	.37	.15***	.28	.14***
3	PA	.35	.02	.39	.02	.29	.01
2	PA,OA	.26	.10**	.29	.07**	.16	.02
3	MA	.35	.09**	.39	.10***	.29	.13***

Note. IQ = Raven’s Standard Progressive Matrices (raw scores); MA = morphological awareness (including HPA = Homophone awareness; CWP = Compound word production; HGA = Homograph awareness); PD = phonological awareness (including SD = Syllable deletion; PD = Phoneme deletion); OA = orthographical awareness.

*** *p*<. 001;

** p<. 01.

### Test of the mediation model

A series of structural equation modeling (SEM) analyses were conducted using the statistical software AMOS 17.0 to explore whether reading fluency mediated the effect of metalinguistic awareness on reading comprehension after controlling for IQ. All the predictor measures were allowed to correlate with each other. For all grades, because there was only a direct effect between morphological awareness and reading comprehension in regression analysis, the mediated models that included a mediated path was conducted. The good model fit was indicated by 1) CFI (comparative fit index) values, TLI (Tucker-Lewis index) values and IFI (incremental fit index) values greater than. 90; 2) RMSEA (root mean square error of approximation) values less than or equal to. 08 [62]; 3) *x* ^*2*^/*df* values less than 3. The Beta weights with significant probability level in the path models of four grades were reported in Table 5. The fit indexes of four mediation models were shown in Table 6.

**Table 5:** The path models for all grades.

Grade	Dependent variables	Indicators	Estimate	Error	C.R.	***p*** value	Beta weight
**G2**	Reading fluency	MA	46.46	8.96	5.18	.00	.72
	Reading comprehension	Reading fluency	.01	.002	2.99	.00	.32
		MA	.32	.14	2.21	.03	.31
		IQ	.06	.03	1.98	.05	.16
**G3**	Reading fluency	MA	58.01	13.56	4.28	.00	.60
	Reading comprehension	Reading fluency	.002	.001	2.06	.03	.19
		MA	.46	.15	3.02	.00	.41
		IQ	.09	.028	3.23	.00	.25
**G4**	reading fluency	MA	19.13	9.59	1.99	.05	.23
	Reading comprehension	Reading fluency	.01	.00	2.44	.02	.18
		MA	.37	.11	3.30	.00	.39
		IQ	.09	.03	2.39	.02	.22

**Table 6:** The fit indexes of three models.

	*x* ^*2*^/*df*	CFI	IFI	TLI	RMSEA
Grade 2	1.73	.95	.95	.91	.07
Grade 3	.68	1.00	1.03	1.06	.00
Grade 4	.94	1.00	1.01	1.02	.00

Table 6 indicated the fit indexes of four mediation models, which showed that these models had a good fit. All mediation models were analyzed controlling for IQ value. The mediation models showed that reading fluency partially mediated the effects morphological awareness on reading comprehension in grades 2–4.

## Discussion

This current study showed that only morphological awareness predicted reading comprehension, but phonological awareness and orthographical awareness did not do in grades 2–4. Moreover, reading fluency was assumed to significantly predict reading comprehension, which mediated partially the effect of morphological awareness on reading comprehension in grades 2–4.

### Phonological awareness, morphological awareness, and orthographical awareness predicted reading comprehension after IQ was controlled for

In grades 2–4, morphological awareness uniquely explained 9%, 10% and 13% variance of reading comprehension respectively, controlling for IQ, phonological awareness and orthographical awareness. The amount of variance of reading comprehension explained by morphological awareness increased with the grade level. The results showed morphological awareness contributed to reading comprehension—the reading distant outcome. It was consistent with previous studies [5,31,32] that had shown the importance of morphological awareness for Chinese reading comprehension. Morphological awareness, defined as awareness of and access to morphemes and their structure composed of two or more morphemes, can advance the understanding of words and characters [5]. Children can infer the meanings of novel words and characters according to either known morphemes or rules of morpheme structures and can construct their understanding of text through bottom-up processing.

Apart from the role of morphological awareness, this work also investigated the role of phonological awareness and orthographical awareness on reading comprehension. However, phonological awareness and orthographical awareness did not contribute to reading comprehension. The role of phonology on reading comprehension may be explained by morphological awareness because phonological awareness was closely related to morphological awareness. For grades 2–4, the relation coefficient was 0.78, 0.71, and 0.45, respectively, and was significant in mediate model for all grades. Wang et.al [22] demonstrated that a morpheme was represented by a cluster of sounds, and children must segment the speech stream to identify the meaning that was borne by the speech stream. In addition, the reason may be phonological awareness and orthographical awareness arrived at a higher level in grades 2–4. From Table 1, the syllable deletion (total scores were 10) mean was approximately 9 from grades 2 to 4. Moreover, Table 1 showed that orthographical awareness was not significantly different between grades 3 and 4. Prior study [63] also presented that orthographical awareness reached a higher level in grade 2, and the correct rate of judging position error, disordered stroke and radical error reached more than 80%. The higher level of phonological awareness and orthographical awareness explained little variance in reading comprehension.

### The mediator role of reading fluency between metalinguistic awareness and reading comprehension

Our results indicated that reading fluency mediated the effect of some components of metalinguistic awareness on reading comprehension. In grades 2–4, reading fluency partially mediated the effect of morphological awareness on reading comprehension.

The current findings provided support for the mechanism which was proposed by Yeung et al.[41] and Kuo et al.[42], which suggested that morphological awareness may influence reading comprehension directly and indirectly through quick word reading. At the same time, our research converged with the finding that reading fluency was a mediator between morphological awareness and reading comprehension [1]. In the introduction, the plausible explanations of direct contributions of morphological awareness to reading comprehension had been discussed. Next, there was the plausible explanation for the mediating role of reading fluency between morphological awareness and reading comprehension in grades 2–4.

Morphological processing may advance the accurate retrieve and integration of character meaning so that it speeded up the sentence reading [1], and thereby reading sentences smoothly saved the sources for passage comprehension [17,18]. This explanation is consistent with the prior study which reading fluency linked word recognition to reading comprehension [10,57,64]. There are many homophones and homographs in Chinese. Homophone awareness and homograph awareness influenced the accurate reading of characters [65], and reading accuracy was one component of fluency [43], so morphological awareness contributed to one main component of fluency. Reading accuracy may decrease the time spent on reading error words, in turn to increase reading comprehension. In addition, students with more developed awareness of compound word production may be easier to construct the meaning of words [12] and to parse the syntactic structures of connected phrases and sentences effortlessly [1], thereby, yielding more efficient reading comprehension. This result also proved that morphology may be an important component of fluency from the multidimensional view [10,11].

To summarize, reading fluency could play a partial mediator role between morphological awareness and reading comprehension in grades 2–4.

### Implication

This study demonstrated that certain aspects of metalinguistic awareness do not only contribute directly to reading comprehension in elementary grades, but contribute to it indirectly through reading fluency. So, in instruction, teachers should strengthen the ability of speed-reading as well as morphological awareness. Quickly reading and accessing word meanings are necessary for reading comprehension. Very fine visual content of text [26] and clear visual cues to word boundaries [66] can improve actual word identification and produce faster reading times, and repeated reading is a good method to increase the speed of reading [58]. The improvement of reading fluency would promote reading comprehension.

From the theoretical perspective, this research has verified the lexical quality hypothesis. According to the lexical quality hypothesis [17], reading comprehension relies on the quality of lexical representation, which is determined by the extent of the bonding of orthographic, phonological, and semantic components. From this study results, morphological awareness influenced reading comprehension through the quick accessing of words and reading fluency. Only morphological awareness of components of metalinguistic awareness influenced reading rate,. Among them, morphology might be the core part of high-quality lexical representation. In 2–4 grades, if morphological representation is of high quality and accurate, reading comprehension may be achieved smoothly. So, in the lexical quality hypothesis, morphology could be the key. The roles of reading-related cognitive skills may change with grade level. Additionally, this study showed that morphology may be the important part of fluency according to the multidimensional view [10].

We cannot see the direction of factor contribution because this study is a cross-section design. Only through a longitudinal study can we find the causal relationship among factors. In future studies, we can explore the causal relationship between reading fluency, metalinguistic awareness and reading comprehension through a longitudinal study. In this study, silent reading fluency as the indicator of reading fluency was discussed, lacking oral reading fluency, which can be researched in future study.
